# Human Rights and Inclusion of Vulnerable Groups in Health and Well-Being Policy Documents Relevant to Children and Young People in Ireland

**DOI:** 10.3390/ijerph21091252

**Published:** 2024-09-21

**Authors:** Megan Lambert, Joanne McVeigh

**Affiliations:** 1 Department of Psychology, Maynooth University, W23 F2K8 Maynooth, Ireland; 2 Assisting Living and Learning (ALL) Institute, Maynooth University, W23 F2K8 Maynooth, Ireland

**Keywords:** equity, EquiFrame, human rights, vulnerable groups, social inclusion, policy analysis

## Abstract

Children and young people constitute a structurally vulnerable group who often experience specific barriers when trying to exercise their rights, including the right to health. The aim of this study was to examine core concepts of human rights and inclusion of vulnerable groups in a sample of national health and well-being policy documents relevant to children and young people in Ireland. Using EquiFrame, a validated policy content analysis tool, 3 policy documents were analysed in relation to their commitment to 21 core concepts of human rights and inclusion of 13 vulnerable groups. The *Overall Summary Ranking* of each policy varied: ‘Better Outcomes, Brighter Futures’–Moderate, ‘Sláintecare’–Moderate, and the ‘Wellbeing Policy Statement’–Low. Across all three documents, *Core Concept Coverage* was high while *Core Concept Quality* was low. The findings demonstrate that these documents cover a wide range of human rights but fail to provide specific policy actions to address human rights or an intention to monitor human rights.

## 1. Introduction

### 1.1. Human Rights and Social Inclusion of Children and Young People

Children constitute 30.3% of the global population, 18.3% of the European population, and 23.2% of the Irish population [1,2]. The United Nations Convention on the Rights of the Child (UNCRC) advocates for a range of civil and political rights and economic, social, and cultural rights of children and young people, including the right to health [3,4]. Four general principles underpin the Convention, including non-discrimination (Article 2), the best interest of the child (Article 3), the right to life, survival, and development (Article 6), and the right to be heard (Article 12) [4]. At the European regional level, guidelines for children’s rights are set out in the EU Strategy on the Rights of the Child [1]. This upholds the right of children to life without discrimination and intimidation and access to the same rights as anyone else [1].

However, children and young people constitute a structurally vulnerable group who often experience specific barriers when trying to exercise their rights, including attitudinal, financial, and legal barriers, as well as confidentiality concerns, shame, and fear of being judged, which prevent young people from accessing healthcare [5,6,7,8]. Furthermore, intersectionality may compound barriers to accessing services for children who experience multiple forms of discrimination and marginalisation, such as children who are living in poverty [1,3]. Poverty greatly impacts the right to health of children, for example with respect to the consumption of more unhealthy foods and higher risk of obesity [9,10]. According to the national *Growing Up in Ireland* study (Cohort ‘08), 42% of families who responded to the survey reported having financial difficulty, and 10% of those claimed that they were under extreme financial strain [11]. Poverty is closely associated with poor educational attainment, which also has a profound impact on the health of children and young people [12,13]. Those who have a poorer education are more likely to live in poverty as adults and are also less likely to be able to provide good healthcare and nutrition to their own children [14]. Poverty during childhood has also been found to have detrimental effects on mental health, whereby an increase in depressive symptoms and anxiety has been found in adolescents living in high poverty neighbourhoods [15,16,17,18]. As children constitute a structurally vulnerable group, at risk of double or triple discrimination and marginalisation, it is crucial for policies to be rights-based and to make explicit provisions for the specific needs and particular barriers experienced by children and young people.

### 1.2. Policy Analysis

Policy analysis can be used to evaluate if a policy adopts an evidence-based approach [19] and allows evaluation of a policy to inform policymakers [20,21]. Policy analysis may be used to analyse policy content and/or policy processes, including policy development, implementation, and evaluation, alongside the effects of policies. Policy analysis can therefore be used to systematically review the implementation and impact of any given policy to inform future policy revision and development [22]. Put simply, policy analysis aims “to understand who develops and implements certain policies, for whom, by what, with what effects” [23].

Policy analysis can be used to assess the extent to which human rights and the social inclusion of vulnerable groups are prioritised in a policy. Policies underpinned by the principle of *equity* enable resources and services to be provided to people based on need, ensuring that vulnerable groups are treated as a priority and are not disadvantaged due to barriers to accessing services [24,25,26]. Although policies guided by the principle of *equality* often use universal language such as “for all citizens”, such policies do not acknowledge the specific needs and barriers of particular vulnerable groups [21,27]. As noted by Huss and MacLachlan [28]: “Equality as an outcome requires equity in the process”.

The principles of equity and social inclusion are espoused in the Sustainable Development Goals (SDGs) [29], which call for those who are most in need to be reached first to ensure that vulnerable groups are not forgotten in policies [30]. It is crucial that equitable health is embedded within the content and ethos of any given policy [21,31]. Policies must therefore demonstrate a commitment to the inclusion of all vulnerable groups to ensure that no group is prioritised over the needs of another [21,32].

### 1.3. Research Aim

The aim of this study was to examine core concepts of human rights and inclusion of vulnerable groups in a sample of national health and well-being policy documents relevant to children and young people in Ireland. It is crucial that policies address the specific needs, aspirations, and barriers experienced by children and young people in relation to accessing resources and services. The findings from the evaluation of these policies will provide evidence for policy revision and development underpinned by rights, equity, and social inclusion.

## 2. Materials and Methods

### 2.1. The EquiFrame Framework

While the importance and usefulness of policy analysis is widely recognised, there has been a lack of tools developed in this area [33]. EquiFrame is a policy content analysis tool that was developed to quantitatively assess the extent to which policy content explicitly commits to human rights and the social inclusion of vulnerable groups [24]. The term ‘vulnerable groups’ in this context denotes groups that are structurally vulnerable, rather than intrinsically or inherently vulnerable; the term ‘structural vulnerability’ examines the ways in which economic, social, and political hierarchies give rise to and pattern ill-health [34]. As described by Bourgois et al. [35], a person is structurally vulnerable “when their location in their society’s multiple overlapping and mutually reinforcing power hierarchies (e.g., socioeconomic, racial, cultural) and institutional and policy-level statuses (e.g., immigration status, labor force participation) constrain their ability to access healthcare and pursue healthy lifestyles” (p. 17).

EquiFrame was developed in consultation with a wide range of stakeholders, including academics, non-governmental organisations, clinicians, and members of vulnerable groups, from a wide range of countries including Sudan, Namibia, Malawi, South Africa, Norway, and Ireland [24]. EquiFrame is a validated tool that has been used to analyse more than 70 African health policies, alongside policies across Spain, Ukraine, India, and Ireland [21,24].

It has been used to assess a wide variety of health-related policies such as national HIV and malaria policies [21,29,32,36], mental health policies [21,37], and national health and drug policies [21,31]. Although EquiFrame primarily focuses on health policies, due to the framework’s flexibility it has also been used in a variety of other policy areas such as Irish housing [21,38].

As a policy content analysis tool, EquiFrame examines the degree of commitment of a policy to 21 core concepts of human rights and inclusion of 12 vulnerable groups (please see Table 1 and Table 2 below). EquiFrame uses four indices to quantify the findings from policy analysis [24]:

1. Core Concept Coverage indicates the percentage of core concepts of human rights that are explicitly mentioned in a policy out of the 21 core concepts.
2. Vulnerable Group Coverage indicates the percentage of vulnerable groups that are identified throughout a policy.
3. Core Concept Quality indicates the percentage of core concepts that are scored either as 3 or 4 out of the 21 core concepts:
  1—Core concept only mentioned.
  2—Core concept mentioned and explained.
  3—Specific policy actions identified to address the concept.
  4—Intention to monitor core concept expressed.
4. Finally, each policy is given an Overall Summary Ranking of either *Low* (policy scored < 50% on two or three of the indices above), *Moderate* (policy scored ≥ 50% on two of the indices), or *High* (the policy scored ≥ 50% on all three indices).

### 2.2. Modifications to the Framework for the Current Study

EquiFrame is a flexible framework that allows for some vulnerable groups or core concepts to be added or excluded from a study, as determined by the particular culture and context; despite this, any revisions must be well-grounded in the literature and human rights documents [21,35]. For the purposes of the present study, adolescents who identify as lesbian, gay, transgender, queer, or other inclusive terms (LGBTQ+) were included as an additional vulnerable group. It is crucial to examine the extent to which LGBTQ+ adolescents are included in policies, as they often face several barriers to health services and education services [39,40].

The most prominent barrier that LGBTQ+ youth face when trying to access services is discrimination, including microaggression [41]. Microaggression denotes brief verbal or behavioural actions, either intentional or unintentional, which can result in hostile or derogatory communication towards a person [41,42]. For example, a study on reported emergency department avoidance, use, and experiences of transgender persons in Ontario, Canada, found that trans-specific negative experiences in the emergency department were reported by 52% of users presenting in their felt gender [40]. The study also reported that 21% had avoided the emergency department on at least one occasion due to concerns surrounding their gender identity [40]. It is therefore crucial to include LGBTQ+ youth as an additional vulnerable group to ensure that policies take into consideration the additional barriers and discrimination that often prevent this group from accessing services.

### 2.3. Selection of Policies

The three policy documents that were selected for this study were relevant to children’s rights in Ireland, including their right to health and education. All policy documents were freely accessible and were the most recent versions of the policy documents at the time of the study. The following documents were selected for this study:

1. ‘Better Outcomes, Brighter Futures: The National Policy Framework for Children and Young People, 2014–2020’. This policy framework was developed by the Department of Children, Equality, Disability, Integration and Youth. ‘Better Outcomes, Brighter Futures’ takes an overall approach to children’s rights including sections that focus on health. The policy framework outlines the government’s commitments to children and young people up to the age of 24 years. The purpose of the framework is to identify areas that, given specialised attention, can greatly improve outcomes for children and young people, and to improve the effectiveness of pre-existing policies and resources.
  The framework adopts an outcomes-based approach in which five national outcomes are identified, to be achieved by focusing on six transformational goals across a seven-year period. The framework also contains a list of committees and groups that are responsible for the implementation of the framework: the Cabinet Committee on Social Policy, Children and Young People’s Policy Consortium, Advisory Council, Implementation Team, and National Strategies by Age + Priority Areas.
  The framework is 168 pages in total, including a section on each of the six Transformational Goals and the five National Outcomes. It also includes a section on how the government intends to implement the framework, including measuring progress and ensuring accountability.
2. ‘Sláintecare Implementation Strategy and Action Plan 2021–2023’ was developed by the Department of Health. Sláintecare is Ireland’s plan and strategy for reform of the health and social care system, which aims to provide equitable healthcare across the country. It focuses on healthcare and the right to health for the entire Irish population, including children. It was initially developed to address the on-going effects that COVID-19 had on the healthcare system and to also accommodate the growing and aging population of Ireland.
  The strategy outlines a number of Fundamental Principles: Patient is Paramount, Timely Access, Prevention and Public Health, Free at the Point of Delivery, Workforce, Public Money and Interest, Engagement, and Accountability. It contains 72 pages covering the Reform Programme 1 and Reform Programme 2, Reform Programme Implementation, and a Strategic Action Plan Development SWOT Analysis and Risk Assessment. It states that progress reports and updates will be given to the Cabinet Committee on Health and the Joint Oireachtas Committee on Health.
3. The ‘Wellbeing Policy Statement and Framework for Practice 2018–2023’ was also analysed in this study. The policy statement was developed by the Department of Education and Skills and looks at children’s overall wellbeing, including physical and mental health. The policy statement focuses on promoting well-being for children and young people in schools and centres for education.
  The policy statement follows a number of key principles to ensure that well-being is promoted within the education system. Key principles set out in the policy statement include: Child/young person-centred; equitable, fair and inclusive; evidence-informed; outcomes focused; and partnership/collaboration. The document is 56 pages in total, containing an explanation for the need for a Wellbeing Policy Statement, a description of what well-being means, the roles of both schools and government, and a section on the implementation of the policy statement.

## 3. Results

Table 3 provides a summary of results across all three policies for EquiFrame’s summary indices, including Vulnerable Group Coverage, Core Concept Coverage, Core Concept Quality, and the Overall Summary Ranking. The findings for each policy are discussed in more detail below.

### 3.1. Better Outcomes, Brighter Futures Policy Framework

#### 3.1.1. Vulnerable Group Coverage

For this policy, all 13 vulnerable groups were identified at least once, resulting in a score of 100% for Vulnerable Group Coverage. The most frequently mentioned vulnerable groups were *Youth* (865 times), *Children (with special needs)* (41 times), *Disabled persons* (27 times), *Persons with Limited Resources* (26 times), *Ethnic Minorities* (15 times), and *Displaced Populations* (12 times). Other vulnerable groups that were explicitly mentioned included: *Suffering from Chronic Illness* (7 times), *LGBTQ+ Community* (5 times), *Living Away from Services* (3 times), and *Increased Relative Risk for Morbidity* (twice). The remaining vulnerable groups of *Mother Child Mortality*, *Women Headed Household*, and *Aged*, all only mentioned once, are those that were mentioned the least throughout this policy.

#### 3.1.2. Core Concept Coverage

In total, 20 core concepts were mentioned at least once throughout this policy, resulting in a Core Concept Coverage score of 95%. The core concept of *Entitlement* was the only concept that was not mentioned. The most frequently mentioned core concept was *Participation* (49 times), followed by *Co-ordination of Services* (48 times), *Quality* (35 times), and *Family Resource* (30 times). The following core concepts were mentioned the least: *Liberty* (twice), *Autonomy* (twice), *Privacy* (twice), and *Cultural Responsiveness* (once).

#### 3.1.3. Core Concept Quality

With regards to Core Concept Quality, 33% of concepts were scored as either 3 or 4. The core concepts of *Non-discrimination*, *Protection from Harm*, *Contribution*, *Prevention*, *Capacity Building*, *Access*, and *Efficiency* were scored at a level 2, as they were only mentioned and explained in the policy. Core concepts that were scored at a level 3 (i.e., specific policy actions were identified to address the concept) included *Participation*, *Co-ordination of Services*, *Integration*, *Family Resource*, *Family Support*, and *Quality*. Only the core concept of *Accountability* was scored at a level 4, namely an intention to monitor the concept was expressed. The remaining core concepts—*Individualised Services*, *Capability Based Services*, *Liberty*, *Autonomy*, *Privacy*, and *Cultural Responsiveness*—were scored as a level 1 (only mentioned).

#### 3.1.4. Overall Summary Ranking

As this policy scored ≥ 50% on two of the three indices, the Overall Summary Ranking for this policy was *Moderate*.

### 3.2. Sláintecare Plan and Strategy

#### 3.2.1. Vulnerable Group Coverage

Across the Sláintecare policy, 9 of the 13 vulnerable groups were mentioned at least once, resulting in a Vulnerable Group Coverage score of 69%. The vulnerable groups of *Aged* (22 times) and those *Suffering from Chronic Illness* (13 times) were mentioned the most frequently, followed by *Persons with Limited Resources* (4 times) and *Mother Child Mortality* (4 times). The vulnerable groups of *Women Headed Household*, *Youth*, and *Disabled* were each mentioned twice, and the vulnerable groups of *Ethnic Minorities* and those *Living Away from Services* were only mentioned once. The following vulnerable groups were not identified in this policy: *Increased Relative Risk for Morbidity*, *Children (with special needs)*, *Displaced Populations*, and *LGBTQ+ Community.*

#### 3.2.2. Core Concept Coverage

The Sláintecare policy received a Core Concept Coverage score of 76%, as it mentioned 16 of the 21 core concepts. The most frequently mentioned core concept was *Prevention* (41 times), followed by *Efficiency* (24 times), *Capacity Building* (18 times), *Family Support* (15 times), *Quality* (15 times), *Accountability* (12 times), *Entitlement* (11 times), *Co-ordination of Services* (11 times), *Liberty* (10 times), and *Access* (10 times). Further core concepts that were mentioned included: *Participation* (7 times), *Individualised Services* (6 times), *Autonomy* (5 times), *Protection from Harm* (4 times), *Integration* (3 times), and *Contribution* (3 times). The core concepts of *Non-discrimination*, *Capability Based Services*, *Privacy*, *Family Resource*, and *Cultural Responsiveness* were not identified in the policy.

#### 3.2.3. Core Concept Quality

The Sláintecare policy scored 29% for Core Concept Quality, with 6 of the 21 core concepts scored either as 3 or 4. The core concepts of *Family Support* and *Accountability* scored the highest at a level 4, i.e., an intention to monitor was expressed. A score of level 3 (specific policy actions were identified) was given to the following core concepts: *Protection from Harm*, *Access*, and *Efficiency*. Core concepts that were scored at a level 2 (mentioned and explained) included: *Entitlement*, *Participation*, *Co-ordination of Services*, *Contribution*, *Prevention*, and *Capacity Building*. The remaining core concepts that were mentioned in the policy were scored at a level 1 (only mentioned), including *Individualised Services*, *Liberty*, *Autonomy*, *Integration*, and *Quality*.

#### 3.2.4. Overall Summary Ranking

As the policy scored ≥ 50% on two of the three indices, the Overall Summary Ranking for this policy was *Moderate*.

### 3.3. Wellbeing Policy Statement and Framework for Practice

#### 3.3.1. Vulnerable Group Coverage

The Wellbeing Policy Statement scored 46% for Vulnerable Group Coverage, with 6 of the 13 vulnerable groups being identified throughout the policy. The vulnerable group of *Youth* (286 times) was the most frequently mentioned, followed by *Disabled* (14 times), and *Children (with special needs)* (13 times). The remaining vulnerable groups of *Persons with Limited Resources*, *Ethnic Minorities*, and the *LGBTQ+ Community* were each only mentioned once throughout the policy. The following vulnerable groups were not identified in the policy: *Increased Relative Risk for Morbidity*, *Mother Child Mortality*, *Women Headed Household*, *Aged*, *Displaced Populations*, *Living Away from Services*, and *Suffering from Chronic Illness*.

#### 3.3.2. Core Concept Coverage

This policy received a Core Concept Coverage score of 81%, with 17 of the 21 core concepts being identified. The most frequently mentioned core concepts included: *Capacity Building* (11 times) and *Co-ordination of Services* (10 times). Other core concepts that were mentioned included: *Access* (7 times), *Individualised Services* (7 times), *Contribution* (7 times), *Prevention* (5 times), *Quality* (4 times), and *Family Resource* (3 times). Several core concepts were mentioned twice throughout the policy, including *Capability Based Services*, *Participation, Accountability*, and *Integration*. The core concepts of *Efficiency*, *Autonomy*, *Protection from Harm*, *Entitlement*, and *Family Support* were mentioned only once in the policy. The remaining core concepts were not mentioned: *Non-discrimination*, *Cultural Responsiveness*, *Liberty*, and *Privacy*.

#### 3.3.3. Core Concept Quality

In total, 2 of the 21 core concepts were scored at a level 3 or 4 in terms of quality, affording the policy a score of 10% for Core Concept Quality. The core concepts of *Individualised Services* and *Protection from Harm* were the only concepts scored at a level 3, i.e., specific policy actions were identified. The following core concepts were scored at a level 2 (mentioned and explained): *Co-ordination of Services*, *Family Resource*, *Capacity Building*, and *Access.* A score of level 1 (only mentioned) was given to the remaining identified core concepts, i.e., *Entitlement*, *Capability Based Services*, *Participation*, *Autonomy*, *Integration*, *Contribution*, *Family Support*, *Accountability*, *Prevention*, *Quality*, and *Efficiency.*

#### 3.3.4. Overall Summary Ranking

As this policy scored < 50% on two of the indices above, the Overall Summary Ranking was *Low.*

## 4. Discussion

The policies analysed in this study are three of the main policies in Ireland today that address the rights of children and young people in Ireland. An Overall Summary Ranking of *Moderate* was given to two of the three policies included in this analysis, ‘Better Outcomes, Brighter Futures’ and ‘Sláintecare’, while the ‘Wellbeing Policy Statement’ scored as *Low*. It is concerning that these policies have scored *Moderate to Low* in light of their impact on the lives of children and young people in Ireland. If national policies are not rights-based and underpinned by the ethos of inclusion and equity, it is less likely that these values will be embodied in service delivery.

### 4.1. Core Concept Coverage and Core Concept Quality

All three policies surpassed EquiFrame’s criterion of 50% for Core Concept Coverage. The core concept of *Access* was mentioned in all three policies. Access to services, often linked to being part of a vulnerable group, is often one of the first barriers people face when trying to avail of a service [39,40,43]. Numerous barriers can impact access to health services, including discrimination, difficulties in communication, lack of information, and inadequate infrastructure for wheelchair access [44]. Although the core concept of *Access* was mentioned in all policies, the highest score for Core Concept Quality that it received in any policy was 2 (core concept mentioned and explained). Although *Access* was therefore mentioned in all policies, none of the policies outlined specific policy actions to address access or expressed an intention to monitor access.

The core concept of *Non-discrimination* was only mentioned in ‘Better Outcomes, Brighter Futures’ and scored as 2 (core concept mentioned and explained) with regards to Core Concept Quality. Each person who avails of a service, in particular health services, deserves to attend without fear of experiencing discrimination due to identifying with a vulnerable group [40]. For example, in a poll conducted on discrimination by UNICEF of over 407,000 young people, the ‘U-Report’ found that 63% of participants had experienced discrimination within their daily environment including schools and the workplace [45]. National origin (20%), age (17%), and gender identity (15%) were reported as the most common reasons for discrimination. For children, their main source of socialisation is school; the attitudes and expectations of their teachers and peers therefore greatly impacts their participation and outcomes in education. A study conducted in the UK found that Eastern European students reported experiencing low expectations from their teachers, and Indigenous children in Australia were found to experience more bullying and racial discrimination compared to their Australian peers [46,47]. This effect on a child’s education can result in a higher likelihood of incarceration and lower employment rates in adulthood, further leading to poorer health and nutrition [45].

Continuous exposure to discrimination is considered a source of trauma and has been found to lead to poor mental health, including emotional and behavioural difficulties in children [48,49]. In adolescence, discrimination has also been found to increase cortisol levels, which in turn has detrimental health effects such as anxiety, depression, and increased blood pressure [50]. National policies must therefore explicitly state how the right to non-discrimination will be upheld, to prevent discrimination within a service, either by staff or by other service users. It is crucial that policies contain specific policy actions and an intention to monitor the right to non-discrimination.

Although Core Concept Coverage was high across all policies, it is alarming that Core Concept Quality was below 50% in each policy. The core concept of *Autonomy* was scored as 1 in all three policies, while *Capacity Building* was scored at a level 2 across all three policies and *Quality* was scored at a level 2 in *Sláintecare* and the *Wellbeing Framework*. Mentioning these core concepts may have very little impact if they are not adequately addressed. For example, simply stating that the service should provide “higher quality” care is not sufficient. It is crucial for policies to provide for specific policy actions to address human rights and to express an intention to monitor human rights.

### 4.2. Vulnerable Group Coverage

Two of the policies scored above 50% for Vulnerable Group Coverage, with ‘Better Outcomes, Brighter Futures’ mentioning all 13 vulnerable groups. The vulnerable groups of *Persons with Limited Resources*, *Youth*, *Ethnic Minorities,* and *Disabled* were mentioned to some degree across all three policies. However, the group *Ethnic Minorities* was mentioned only once in both Sláintecare and the *Wellbeing Policy*.

Persons with disabilities were mentioned (27 times) in ‘Better Outcomes, Brighter Futures’, while also acknowledging the different presentations of disabilities such as special educational needs, mental health difficulties, physical disability, and behavioural difficulties. However, the vulnerable group of *Disabled* persons was only mentioned twice in *Sláintecare*. With 6.4% of children in Ireland having a disability, it is crucial that this vulnerable group is acknowledged in state policies [51]. A significant barrier for those with disabilities is often lack of awareness of disabilities and communication differences [52]. A lack of knowledge about disabilities may lead to healthcare staff exhibiting poor attitudes towards patients and to misunderstanding the needs of patients with disabilities [52]. Lack of knowledge and understanding may also result in inadequate communication, resulting in poor service provision [52,53,54].

While protection of the rights of all children is imperative, children who experience further vulnerability due to factors such as poverty and/or disability may experience increased barriers to accessing services and resources. For example, the vulnerable group of *Children (with special needs)* was mentioned frequently in both ‘Better Outcomes, Brighter Futures’ (41 times) and the ‘Wellbeing Policy Statement’ (13 times). However, in Sláintecare, *Children (with special needs)* was not mentioned at all. This finding was expected as ‘Better Outcomes, Brighter Futures’ and the ‘Wellbeing Policy Statement’ are focused specifically on children and young people. Nonetheless, children and young people need to be explicitly acknowledged to a greater extent in ‘Sláintecare’ as the State’s strategy for reform of the health and social care system, particularly children with increased vulnerability. Children (with special needs) experience an increased risk of health issues, such as increased risk of asthma or bronchitis due to damp and cold home environments [55,56,57,58].

The vulnerable groups of Mother Child Mortality, Women Headed Households, Living Away from Services, Increased Relative Risk for Morbidity, Aged, Displaced Minorities, LGBTQ+, and Suffering from Chronic Illness were not mentioned in the ‘Wellbeing Policy Statement’. It is crucial for the specific needs and barriers of particular vulnerable groups to be explicitly addressed in policies. For example, a study examining the experiences of parents with children with chronic fatigue syndrome/myalgic encephalomyelitis (CFS/ME) found that the main barriers to accessing healthcare services included healthcare workers’ lack of knowledge about the condition, alongside negative attitudes and beliefs towards the condition amongst healthcare professionals [59]. Parents reported that doctors had a negative attitude towards the condition and would often dismiss their child’s symptoms [59].

The inclusion of vulnerable groups is a key human rights value and central to the principle of equity. A complementary perspective is that of ‘proportionate universalism’, which proposes that focusing only on the most vulnerable groups in society will not adequately ameliorate health disparities. Instead, to address the social gradient in health, actions should be universal, but with an intensity and scale that is proportionate to the degree of disadvantage [60,61]. In this regard, to reduce the social gradient, both a universalism approach and a focus on particular disadvantaged groups is needed. Applying this approach requires examining the sections of society that are most disadvantaged and in what ways, and then targeting barriers to resources and services for such groups. To do this in policy instruments, EquiFrame provides a mechanism for identifying those who are not adequately addressed in the content of policies.

### 4.3. Limitations

This analysis focused solely on policy content and did not, therefore, evaluate the inclusiveness or effectiveness of policy processes, including policy development, implementation, and evaluation. While it is important for the content of policies to be inclusive and rights-based, without effective policy implementation, such values are not effectuated in the delivery of services and distribution of resources. It is crucial for both policy content and policy processes to be inclusive and to reflect the lived experience of marginalised groups [62]. In this regard, it is important to examine both policy “on the books” and policy “on the streets” [63]. Although beyond the scope of this study, it is recommended that future research assess the inclusiveness of policy processes using a complementary tool to EquiFrame, the EquIPP framework, which provides a tool to examine equity and social inclusion in the processes of policy development, implementation, and evaluation [62].

It is also important to note that this study focused on just three policy documents relevant to children and young people in Ireland. Due to the interdependence of human rights and the importance of intersectoral coordination within governments, it is recommended for future studies to examine other national policies that may impact on the lives of children and young people in Ireland to determine the extent to which they are underpinned by human rights and grounded in the principles of equity and social inclusion.

The EquiFrame manual defines ‘disabled’ as “[r]eferring to persons with disabilities, including physical, sensory, intellectual or mental health conditions, and including synonyms of disability”, a definition that reflects a medical model of disability. It is important to recognise, however, that disability arises from both individual and contextual causes, whereby people are disabled by both their bodies and by society, a perspective that traverses both the medical and social models of disability [64,65]. This perspective is espoused in the World Report on Disability [66], which defines disability as an “umbrella term for impairments, activity limitations and participation restrictions, referring to the negative aspects of the interaction between an individual (with a health condition) and that individual’s contextual factors (environmental and personal factors)” (p. 4).

## 5. Conclusions

While each of the policies analysed demonstrated good coverage of core concepts of human rights, as reflected in the high Core Concept Coverage scores, all policies received low scores for Core Concept Quality. The findings demonstrate that these documents cover a wide range of human rights but fail to explicitly mention particular human rights or to provide specific policy actions to address rights or an intention to monitor rights. Policies for children and young people in Ireland are needed that are explicitly committed to a broad spectrum of human rights and that are inclusive of all vulnerable groups. Without doing so, policies may prioritise some groups above others, including the needs of dominant groups, while further marginalising particular vulnerable groups [67,68]. To respect, protect and fulfil rights for children and young people in Ireland, urgent policy revision is therefore required.

While coverage of vulnerable groups was high in two policies, less than 50% of vulnerable groups was mentioned in the ‘Wellbeing Policy Statement’. Furthermore, only the ‘Better Outcomes, Brighter Futures’ policy scored 100% for the inclusion of all 13 vulnerable groups. It is crucial that the specific needs and barriers faced by structurally vulnerable groups are acknowledged in policies. The findings from the evaluation of these policies demonstrate that urgent policy revision and development are required to ensure that policies that impact on the lives of children and young people in Ireland are underpinned by human rights, equity, and social inclusion.

## Figures and Tables

**Table 1:** EquiFrame’s Core Concepts of Human Rights [24].

No	Core Concept	Key Question	Key Language
1.	**Nondiscrimination**	Does the policy support the rights of vulnerable groups with equal opportunity in receiving health care?	Vulnerable groups are not discriminated against on the basis of their distinguishing characteristics (i.e., Living away from services; Persons with disabilities; Ethnic minority or Aged).
2.	**Individualized Services**	Does the policy support the rights of vulnerable groups with individually tailored services to meet their needs and choices?	Vulnerable groups receive appropriate, effective, and understandable services.
3.	**Entitlement**	Does the policy indicate how vulnerable groups may qualify for specific benefits relevant to them?	People with limited resources are entitled to some services free of charge or persons with disabilities may be entitled to respite grant.
4.	**Capability based Services**	Does the policy recognize the capabilities existing within vulnerable groups?	For instance, peer to peer support among women headed households or shared cultural values among ethnic minorities.
5.	**Participation**	Does the policy support the right of vulnerable groups to participate in the decisions that affect their lives and enhance their empowerment?	Vulnerable groups can exercise choices and influence decisions affecting their life. Such consultation may include planning, development, implementation, and evaluation.
6.	**Coordination of Services**	Does the policy support assistance of vulnerable groups in accessing services from within a single provider system (interagency) or more than one provider system (intra-agency) or more than one sector (intersectoral)?	Vulnerable groups know how services should interact where inter-agency, intra-agency, and inter-sectoral collaboration is required.
7.	**Protection from** **Harm**	Vulnerable groups are protected from harm during their interaction with health and related systems	Vulnerable group are protected from harm during their interaction with health and related systems
8.	**Liberty**	Does the policy support the right of vulnerable groups to be free from unwarranted physical or other confinement?	Vulnerable groups are protected from unwarranted physical or other confinement while in the custody of the service system/provider.
9.	**Autonomy**	Does the policy support the right of vulnerable groups to consent, refuse to consent, withdraw consent, or otherwise control or exercise choice or control over what happens to him or her?	Vulnerable groups can express “independence” or “self-determination”. For instance, person with an intellectual disability will have recourse to an independent third party regarding issues of consent and choice.
10.	**Privacy**	Does the policy address the need for information regarding vulnerable groups to be kept private and confidential?	Information regarding vulnerable groups need not be shared among others.
11.	**Integration**	Does the policy promote the use of mainstream services by vulnerable groups?	Vulnerable group are not barred from participation in services that are provided for general population.
12.	**Contribution**	Does the policy recognize that vulnerable groups can be productive contributors to society?	Vulnerable groups make a meaningful contribution to society.
13.	**Family Resource**	Does the policy recognize the value of the family members of vulnerable groups in addressing health needs?	The policy recognizes the value of family members of vulnerable groups as a resource for addressing health needs.
14.	**Family Support**	Does the policy recognize individual members of vulnerable groups may have an impact on the family members requiring additional support from health services?	Persons with chronic illness may have mental health effects on other family members, such that these family members themselves require support.
15.	**Cultural** **Responsiveness**	Does the policy ensure that services respond to the beliefs, values, gender, interpersonal styles, attitudes, cultural, ethnic, or linguistic aspects of the person?	(i) Vulnerable groups are consulted on the acceptability of the service provided (ii) Health facilities, goods and services must be respectful of ethical principles and culturally appropriate, i.e., respectful of the culture of vulnerable groups
16.	**Accountability**	Does the policy specify to whom, and for what, services providers are accountable?	Vulnerable groups have access to internal and independent professional evaluation or procedural safeguard.
17.	**Prevention**	Does the policy support vulnerable groups in seeking primary, secondary, and tertiary prevention of health conditions?	
18.	**Capacity** **Building**	Does the policy support the capacity building of health workers and of the system that they work in addressing health needs of vulnerable groups?	
19.	**Access**	Does the policy support vulnerable groups—physical, economic, and information access to health services?	Vulnerable groups have accessible health facilities (i.e., transportation; physical structure of the facilities; affordability and understandable information in appropriate format).
20.	**Quality**	Does the policy support quality services to vulnerable groups through highlighting the need for evidence-based and professionally skilled practice?	Vulnerable groups are assured of the quality of the clinically appropriate services.
21.	**Efficiency**	Does the policy support efficiency by providing a structured way of matching health system resources with service demands in addressing health needs of vulnerable groups?	

**Table 2:** EquiFrame’s Definitions for Vulnerable Groups [24].

	Vulnerable Group	Attributes or Definitions
1.	**Limited Resources**	Referring to poor people or people living in poverty.
2.	**Increased Relative Risk For Morbidity**	Referring to people with one of the top ten illnesses, identified by WHO, as occurring within the relevant country.
3.	**Mother Child Mortality**	Referring to factors affecting maternal and child health (0–5 years).
4.	**Women Headed Household**	Referring to households headed by a woman.
5.	**Children (with special needs)**	Referring to children marginalized by special contexts, such as orphans or street children.
6.	**Aged**	Referring to older age.
7.	**Youth**	Referring to younger age, without identifying gender.
8.	**Ethnic Minorities**	Referring to non-majority groups in terms of culture, race, or ethnic identity.
9.	**Displaced Populations**	Referring to people who, because of civil unrest or unsustainable livelihoods, have been displaced from their previous residence.
10.	**Living Away from Services**	Referring to people living far from health services, either in time or distance.
11.	**Suffering from Chronic Illness**	Referring to people who have an illness that requires continuing need for care.
12.	**Disabled**	Referring to persons with disabilities, including physical, sensory, intellectual, or mental health conditions, and including synonyms of disability.

**Table 3:** Summary of Indices Across All Policies Analysed.

	Vulnerable Group Coverage	Core Concept Coverage	Core Concept Quality	Overall Summary Ranking
Better Outcomes, Brighter Futures	100%	95%	33%	Moderate
Sláintecare	69%	76%	29%	Moderate
Wellbeing Policy Statement	46%	81%	9%	Low

## Data Availability

To obtain further details on the analysis reported in this study, please contact the authors.
